# Empowering Rural Adolescents to Build Healthy Skin Habits and Self-Advocacy Through a School-Based Intervention

**DOI:** 10.7759/cureus.115317

**Published:** 2026-08-27

**Authors:** Jasmine Jaing, Samantha C Escobar, Patricia Kate Balatbat, Carolina Quezada

**Affiliations:** 1 School of Osteopathic Medicine, A.T. Still University School of Osteopathic Medicine in Arizona, Mesa, USA; 2 Department of Internal Medicine, A.T. Still University School of Osteopathic Medicine in Arizona, Mesa, USA

**Keywords:** adolescent health, dermatology education, health literacy, preventive medicine, rural health, school-based intervention, skin health, underserved populations

## Abstract

Background

Chronic skin conditions are common among adolescents and are associated with psychosocial distress, stigma, and reduced quality of life. California's Central Valley is home to underserved populations that experience high UV exposure and reduced access to dermatologic care due to physician shortages and limited health literacy. School-based education offers an accessible approach to improving skin health knowledge and preventive behaviors. This study evaluated the impact of a medical student-led skin health education program on dermatologic knowledge, attitudes, self-efficacy, and preventive behaviors among rural high school students.

Methods

A 30-minute interactive skin health education session was delivered by medical students in a rural California high school health sciences program. Content included skin anatomy, acne, sun protection, common dermatologic conditions, and environmental skin exposures. Anonymous surveys were administered before (n=172), immediately after (n=165), and one month after (n=118) the intervention. The intervention assessed knowledge, attitudes, and self-efficacy, with an open-ended reflection at one-month follow-up. Surveys were anonymous so individual responses could not be linked longitudinally, requiring the three time points to be analyzed as independent samples. Knowledge outcomes were analyzed by three investigators using two-proportion z-tests, Likert-scale responses using two-sided Mann-Whitney U tests with tie corrections, and open-ended reflection responses using qualitative content analysis. Statistical significance was defined as p<0.05.

Results

Immediately following the intervention, knowledge significantly improved in acne etiology (p<0.001), skin anatomy (p<0.001), the role of dermatologists (p<0.001), and melanin function (p=0.003). Improvements in acne etiology and the role of dermatologists remained significant at one month. At one-month follow-up, participants reported more favorable attitudes toward acne (p=0.001) and sunscreen use (p=0.001). Self-efficacy also improved with greater confidence in caring for their skin (p=0.001) and who to seek help from (p<0.001). Among respondents to the one-month reflection, 101 (88.6%) of participants reported adopting at least one new skin health behavior, with increased use of sun protection being the most reported behavior change in 90 (78.9%) participants.

Conclusion

A brief medical student-led skin health education program significantly improved dermatologic knowledge, attitudes, self-efficacy, and preventive health behaviors among rural adolescents. These findings suggest that school-based educational interventions can empower adolescents to take an active role in their health and may serve as an effective strategy for promoting prevention and health literacy in underserved communities.

## Introduction

Chronic dermatologic conditions such as acne and eczema affect a substantial proportion of adolescents and are associated with significant psychosocial burden. Acne vulgaris has been linked to increased rates of anxiety and depression, with meta-analytic evidence suggesting that approximately 22% of affected youth experience comorbid mental health symptoms [1]. In addition to psychological distress, visible skin disease can contribute to stigmatization, bullying, and reduced quality of life among pediatric populations [2]. These social and emotional consequences highlight the importance of early education and timely access to dermatologic care during adolescence.

Despite the high prevalence of dermatologic conditions during adolescence, disparities in dermatologic care persist across socioeconomic, racial, and geographic groups. National analyses demonstrate that access to outpatient dermatology services varies significantly by demographic and socioeconomic characteristics, with underserved populations experiencing reduced access to specialty care [3]. Pediatric populations from minority and low-income communities are disproportionately affected by these barriers, which may contribute to delays in diagnosis, limited preventive education, and worse dermatologic outcomes [4].

These disparities are particularly relevant in rural regions such as California’s Central Valley, where communities face a combination of structural barriers to healthcare access and environmental risk factors. The region is home to large agricultural populations who spend significant time outdoors and experience high levels of ultraviolet exposure. At the same time, shortages of dermatology providers and limited access to specialty care create gaps in preventive dermatologic education and early intervention [3,4].

School-based health education programs provide a promising strategy to address these disparities. The World Health Organization’s Health Promoting Schools framework demonstrates that health education delivered within school settings can improve students’ health knowledge, behaviors, and overall well-being [5]. Schools are uniquely positioned to deliver preventive health interventions to large adolescent populations during critical stages of behavioral development [6].

Dermatology-focused educational interventions delivered in school settings have led to similar benefits. Systematic reviews of sun safety education programs show improvements in students’ knowledge, attitudes, and preventive behaviors [7]. Peer-led dermatology education initiatives such as the Sun Protection Outreach Teaching by Students (SPOTS) program have also demonstrated measurable improvements in skin cancer awareness and prevention practices among adolescents [8].

The present study evaluated the impact of a medical student-led skin health education program delivered to high school students in rural Central California. The intervention aimed to improve dermatologic knowledge, attitudes toward skin health, self-efficacy, and preventive health behaviors.

## Materials and methods

Study design and setting

This interventional study was conducted from January 30, 2026 through March 13, 2026 at Orosi High School, a public high school in Tulare County in California’s Central Valley. The school serves 1,125 students in grades 9-12, of whom 95.5% identify as Hispanic, reflecting the region’s predominantly rural and agricultural population [9]. 

The intervention was evaluated using a single-group design without a control or comparison group. Participant responses were compared across surveys administered at three time points: immediately before the educational session (pre-intervention), immediately following the session (post-intervention), and one month after the intervention. Surveys assessed participant knowledge gains and changes in attitudes and health-seeking behaviors.

Participants

Participants were students enrolled in the Career Pathway Academy of Health Sciences, a specialized academic pathway for students interested in health-related careers. The program has an enrollment of over 300 students and provides exposure to healthcare professions and career development opportunities through a curriculum spanning grades 9-12. 

Students enrolled in participating classes who were present on the day of the intervention were eligible to participate. Survey participation was voluntary and anonymous, and students who declined survey participation were still able to participate in the educational session. A total of 172 students completed the pre-intervention survey, 165 students completed the immediate post-intervention survey, and 118 students completed the one-month follow-up survey to assess knowledge gains and sustained attitudinal changes. Since the survey was offered to all eligible students in participating classes, a prior sample size was not calculated.

Educational intervention

Medical student investigators delivered a 30-minute interactive educational session designed to improve dermatologic health knowledge and preventive behaviors. The educational content was developed using patient and public education resources from the American Academy of Dermatology (AAD) [10]. The curriculum included instruction on basic skin anatomy, causes of common dermatologic conditions such as acne, principles of sun protection, and environmental exposures affecting skin health, like insect and tick bites. 

The session incorporated visual slides, interactive discussion, and opportunities for students to ask questions. All presenters used the same standardized slide deck for each class period to promote consistency in the content delivered across presentations. Intervention fidelity was supported through use of the standardized slide deck, but a formal fidelity assessment was not conducted. Educational content emphasized practical preventive strategies and encouraged students to seek medical care for concerning skin changes or persistent dermatologic symptoms. Each student received a donated skincare item at the end of the intervention, regardless of survey participation.

Survey development and administration 

The survey questions were developed by the study team to assess the educational objectives of the intervention, including dermatologic knowledge, attitudes toward skin health, and self-efficacy related to skin care. The questions were based directly on the educational content presented during the intervention. Faculty reviewed the questions for content, clarity, and relevance. The questionnaire was designed specifically for this educational intervention and was not previously validated. No formal pilot testing, readability assessment, cognitive interviewing, or reliability testing was performed before implementation. Knowledge items consisted of multiple-choice questions addressing skin anatomy, causes of acne, and preventive health practices such as sun protection. Attitudes and self-efficacy were assessed using five-point Likert-scale items ranging from 1 (strongly disagree) to 5 (strongly agree). The complete survey questionnaire is provided in Appendix A.

Participants completed surveys immediately before the intervention, immediately after the intervention, and at one-month follow-up. Surveys were anonymous and did not contain identifiers that permitted responses to be linked across time points. The one-month follow-up survey additionally included an open-ended reflection asking participants to identify one thing they had started doing differently to care for their skin since the intervention.

Statistical analysis

The pre-intervention, immediate post-intervention, and one-month follow-up surveys were analyzed as independent samples because the surveys were anonymous and individual responses could not be linked across study time points. As a result, the data were analyzed to evaluate group-level differences across survey administrations rather than within-participant changes over time. The sample size decreased across survey administration from 172 participants at baseline to 165 immediately post intervention and 118 at one-month follow-up. Therefore, one-month comparisons observed may reflect both effects associated with educational intervention and differences in the composition of respondents who completed each survey. Furthermore, it should be noted that the absence of a control group limits causal attribution of the observed changes to the intervention.

Knowledge outcomes were analyzed using two-proportion z-tests to compare the proportions of correct responses between baseline and immediate post-intervention and between baseline and one-month follow-up. Changes in Likert-scale responses assessing attitudes and health-seeking confidence were evaluated using two-sided Mann-Whitney U tests with tie corrections. Pre- versus post-intervention comparisons assessed immediate changes following the intervention, while pre-intervention versus one-month follow-up comparisons assessed whether differences as a group were present at follow-up. Statistical significance was defined as p<0.05. 

The open-ended reflection question at one-month follow-up was analyzed using qualitative content analysis. Codes were developed from recurring response patterns and organized into categories representing sun-protective behaviors, changes in skincare routines, product awareness, knowledge retention without behavior change, and no reported changes. Initial coding and analysis were done by one investigator. The data were then reviewed by a second investigator for consistency and accuracy. Any discrepancies in coding or categorization were discussed between the two investigators and resolved by consensus. Formal inter-rater agreement statistics were not calculated because responses were not independently coded by both investigators.

Two-proportion z-tests were performed in Microsoft Excel (Microsoft Corp., Redmond, WA, USA) to compare the proportion of correct responses at baseline with the immediate post-intervention and one-month follow-up assessment. Z-values were calculated using pooled standard errors. P-values were derived from the standard normal distribution. Statistical significance was defined as p<0.05. 

Two-sided Mann-Whitney U tests were also calculated in Microsoft Excel to compare baseline responses to immediate post-intervention and one-month follow-up Likert-scale responses. Responses were assigned scores from 1 (strongly disagree) to 5 (strongly agree). For each comparison, responses from the two time points being compared were pooled and ranked according to their Likert-scale scores. If multiple responses had the same Likert score, they were assigned the average rank for that response category. To calculate the baseline rank sum, the average rank for each Likert response category was multiplied by the number of baseline participants who selected that response. These values were then added together to obtain the total baseline rank sum. The sum and sample sizes were then used to calculate the U values for each group. The smaller of the two U values was reported as the Mann-Whitney U value. The expected U value was then calculated from the sample sizes of the two groups. Because the Likert-scale data contained tied responses, the variance of U was adjusted for ties. The standard deviation was calculated from the adjusted variance. Z-values were then calculated by comparing the Mann-Whitney U value with the expected U value and dividing by the standard deviation. P-values were calculated from the corresponding Z-values using the standard normal distribution. Statistical significance was defined as p<0.05. 

Human participant compliance statement

This project was reviewed by the A.T. Still University Arizona Institutional Review Board (IRB), Mesa, AZ, USA (Protocol #2025-135) and determined to be a program evaluation/quality improvement initiative that does not meet the federal definition of human subjects research (45 CFR 46.102). As such, formal IRB approval was not required. All activities were conducted in accordance with institutional policies and ethical standards.

## Results

A total of 172 students completed the baseline survey and were included in the study population. Participants ranged in age from 14 to 18 years and represented students across grades 9 through 12. The majority of students identified as female. Participant characteristics are summarized in Table 1.

**Table 1:** Participant demographics (n=172)

Characteristics	n (%) or value
Total participants	172
Age (years)	14-18
Gender	
Female	143 (83.1)
Male	29 (16.9)
Grade level	
9^th^	48 (27.9)
10^th^	55 (32.0)
11^th^	32 (18.6)
12^th^	37 (21.5)

Knowledge outcomes

Dermatologic knowledge improved following the intervention, with the greatest immediate gains observed in concepts with the lowest baseline knowledge. Knowledge of acne etiology (p<0.001), role of dermatologists (p<0.001), skin anatomy (p<0.001), and melanin function (p=0.003) improved significantly from baseline to immediately post intervention.

At the one-month follow-up, improvements in acne etiology and knowledge of the role of dermatologists remained significantly higher than baseline (both p<0.001), suggesting retention of these concepts over time. Although knowledge of skin anatomy (p=0.059) and melanin function (p=0.115) remained numerically higher than baseline, these differences were no longer statistically significant at one month. This may suggest continued reinforcement of these topics.

No significant changes were observed in knowledge of tick bites, sunscreen use on cloudy days, UV protection, mole warning signs, or insect-borne disease transmission at either immediate post-intervention or one-month follow-up (all p>0.05). Several of these topics, particularly sunscreen use, UV protection, and mole warning signs, had high baseline rates of correct responses, limiting the potential for measurable improvement. Knowledge outcomes are illustrated in Table 2.

**Table 2:** Changes in dermatologic knowledge following the educational intervention Values are presented as n (%), representing the number and percentage of participants who answered each item correctly. Sample sizes were n=172 at baseline, n=165 immediately post intervention, and n=118 at the one-month follow-up. Because individual responses could not be linked across survey time points, samples were analyzed as independent. Two-proportion z-tests were used to compare baseline with immediate post-intervention and baseline with one-month follow-up. Statistical significance was defined as p<0.05, shown in bold font.

Knowledge Item	Pre-intervention n (%)	Post-intervention n (%)	Pre vs. Post z-value	Pre vs. Post p-value	One-Month Follow-up n (%)	Pre- vs. One-month z-value	Pre- vs. One-month p-value
Acne etiology	26 (15.1)	143 (86.7)	13.13	<0.001	64 (54.2)	7.07	<0.001
Role of a dermatologist	91 (52.9)	126 (76.4)	4.50	<0.001	96 (81.4)	4.97	<0.001
Skin anatomy	37 (21.5)	93 (56.4)	6.57	<0.001	37 (31.4)	1.89	0.059
Melanin function	138 (80.2)	151 (91.5)	2.96	0.003	103 (87.3)	1.58	0.115
Tick bite risk	155 (90.1)	150 (90.9)	0.25	0.804	99 (84.0)	-1.58	0.115
Sunscreen on cloudy days	156 (90.7)	154 (93.3)	0.89	0.373	113 (95.8)	1.63	0.102
UV protection	164 (95.3)	160 (97.0)	0.77	0.440	112 (94.9)	-0.17	0.866
Mole warning signs	165 (96.0)	159 (96.4)	0.21	0.836	111 (94.1)	-0.73	0.467
Insect disease transmission	105 (61.0)	96 (58.2)	-0.54	0.592	71 (60.2)	-0.15	0.881

Attitudes toward skin health

Participants demonstrated significant improvements in attitudes toward skin health immediately following the intervention. Agreement that acne is a normal part of adolescence increased (p=0.003), as did perceptions that sunscreen use was worthwhile (p=0.001).

At one-month follow-up, improvements in attitudes toward acne (p=0.001) and sunscreen use (p=0.001) were maintained. Overall, the intervention produced positive differences in attitude that remained significant at one-month follow-up, especially about skin health beliefs related to acne and sun protection. Changes in attitudes are shown in Table 3.

**Table 3:** Changes in attitudes toward skin health following the educational intervention Values represent mean Likert-scale scores (1 = strongly disagree; 5 = strongly agree). Sample sizes were n=172 at baseline and n=165 immediately post-intervention. Total sample size at one month is n=118; however, one participant did not complete this section, resulting in n=117. Pre-intervention versus immediate post-intervention and pre-intervention versus one-month follow-up comparisons were performed using two-sided Mann-Whitney U tests with tie corrections. Because individual responses could not be linked across survey time points, samples were analyzed as independent. U represents the Mann-Whitney U test statistic. Statistical significance was defined as p<0.05, shown in bold font.

Attitude Item	Pre-intervention Mean ± SD	Post-intervention Mean ± SD	Pre vs. Post U	Pre vs. Post p-value	One-month Mean ± SD	Pre vs. One-month U	Pre vs. One-month p-value
Acne is a normal part of growing up	4.22 ± 0.82	4.46 ± 0.71	11,806	0.003	4.51 ± 0.68	8,008	0.001
Wearing sunscreen is worth the effort	4.52 ± 0.68	4.73 ± 0.53	11,855	0.001	4.75 ± 0.52	8,201	0.001
Taking care of my skin is important for my health	4.69 ± 0.57	4.79 ± 0.41	13,282.5	0.168	4.81 ± 0.39	9,245	0.110

Self-efficacy

Significant improvements were observed across measures of self-efficacy immediately following the intervention. Participants reported greater confidence in identifying whom to ask about skin, hair, or nail concerns (p<0.001), greater confidence in caring for their own skin (p<0.001), and increased comfort discussing skin concerns with a healthcare provider or trusted adult (p=0.001). 

At one-month follow-up, improvements were seen in knowing whom to ask about skin concerns (p<0.001) and confidence in personal skin care (p=0.001). Comfort discussing skin concerns also remained higher than baseline, although the difference was no longer statistically significant (p=0.065). Overall, these findings indicate immediate improvement across all three self-efficacy measures, with differences remaining significant at one-month follow-up, as demonstrated in Table 4.

**Table 4:** Changes in self-efficacy and self-advocacy following the educational intervention Values represent mean Likert-scale scores (1 = strongly disagree; 5 = strongly agree). Sample sizes were n=172 at baseline and n=165 immediately post-intervention. Total sample size at one month is n=118; however, one participant did not complete this section, resulting in n=117. Pre-intervention versus immediate post-intervention and pre-intervention versus one-month follow-up comparisons were performed using two-sided Mann-Whitney U tests with tie corrections. Because individual responses could not be linked across survey time points, samples were analyzed as independent. U represents the Mann-Whitney U test statistic. Statistical significance was defined as p<0.05, shown in bold font.

Self-Efficacy Item	Pre-intervention Mean ± SD	Post intervention Mean ± SD	Pre vs. Post U	Pre vs. Post p-value	One-month Mean ± SD	Pre- vs. One-month U	Pre- vs. One-month p-value
I know who to ask if I have questions about skin, hair, or nails	3.61 ± 0.96	4.33 ± 0.67	8,140	<0.001	4.28 ± 0.80	6,091	<0.001
I feel confident in how I take care of my skin	3.57 ± 0.85	4.01 ± 0.86	10,172	<0.001	3.94 ± 0.96	7,619	0.001
I feel comfortable talking with a doctor, nurse, or parent about skin concerns	4.13 ± 0.83	4.41± 0.68	11,503	0.001	4.33 ± 0.71	8,742	0.065

Behavior change

**Figure 1:**
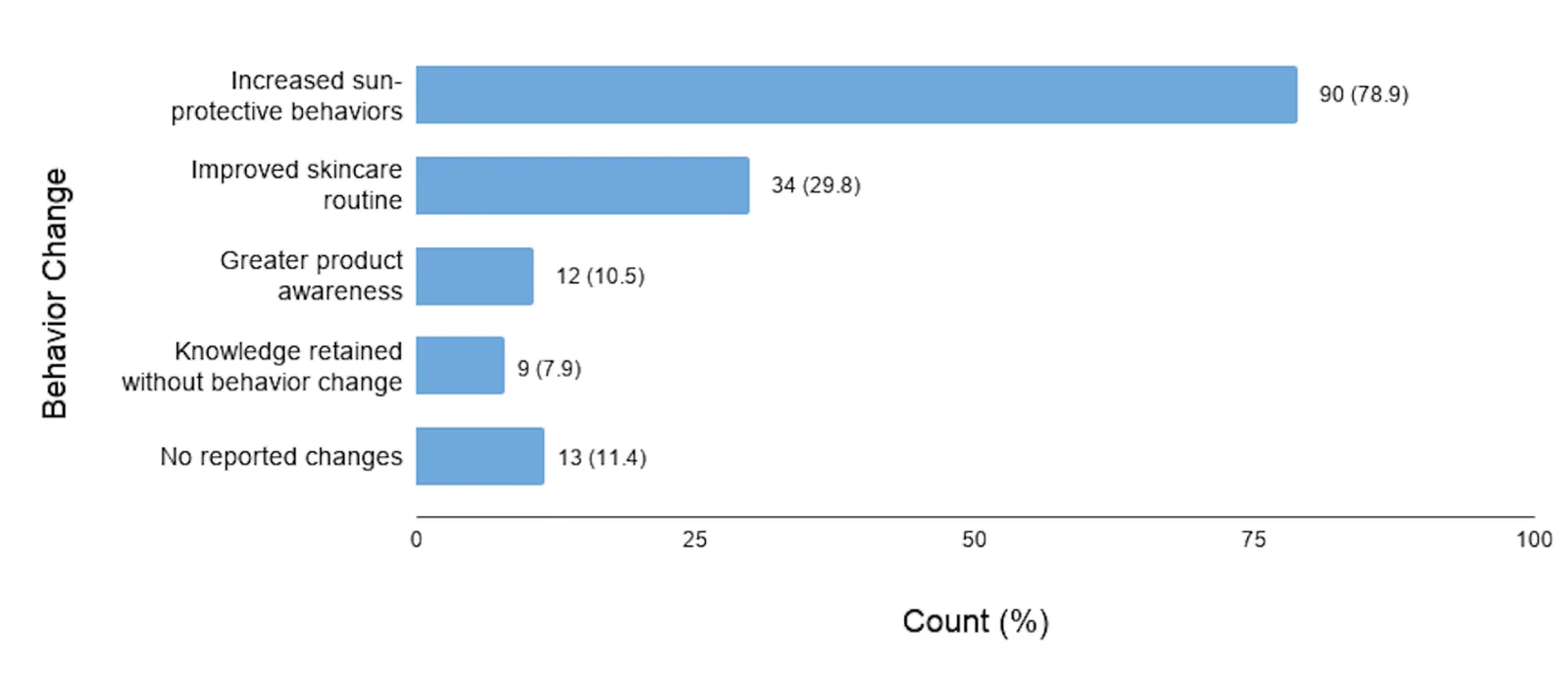
Distribution of self-reported skin health behavior changes among participants one month after the educational intervention Based on qualitative content analysis of open-ended responses from 114 participants. Values are presented as n (%). Categories are not mutually exclusive because participants could report multiple behavior changes within a single response.

Of the 118 participants who completed the one-month follow-up survey, 114 provided responses to the open-ended reflection question. Qualitative content analysis of these responses indicated that 101 (88.6%) of participants reported adopting at least one new skin care behavior, as shown in Figure 1. The most common change was increased sun-protective behavior, reported by 90 (78.9%) participants, including daily sunscreen use, more frequent sunscreen application, or incorporation of sunscreen into their daily routine. Additional changes included improvements in general skincare practices, such as increased cleansing and moisturizing, in 34 (29.8%) participants and greater awareness of appropriate product selection in 12 (10.5%). Responses reflecting knowledge gains without behavioral change were identified in nine (7.9%) participants, while 13 (11.4%) reported no change.

## Discussion

This study extends existing evidence supporting school-based health education by demonstrating that a brief, medical student-led dermatologic intervention can produce sustained improvements in dermatologic knowledge, attitudes, self-efficacy, and health behaviors among rural adolescents. A systematic review of school-based sun protection interventions similarly concluded that educational programs delivered in secondary schools can improve preventive behaviors. It also emphasized the need for higher-quality studies and more comprehensive approaches beyond traditional health education alone [11]. Our findings build upon this literature by demonstrating improvements not only in knowledge but also in attitudes, self-efficacy, and self-reported behavior change following a brief educational intervention.

The intervention was most effective in addressing areas of low baseline knowledge, particularly acne etiology, skin anatomy, and the role of dermatologists. The substantial and sustained improvement in acne-related knowledge is particularly meaningful given the well-established psychosocial burden of acne, which extends beyond physical manifestations. A recent systematic review found that acne is consistently associated with impaired quality of life, reduced self-esteem, anxiety, depression, and social functioning among adolescents and young adults [12]. Additionally, visible skin conditions are known to contribute to stigmatization and reduced quality of life in pediatric populations [2]. By correcting misconceptions and normalizing acne as a common developmental condition, the intervention may help reduce stigma while promoting healthier self-management behaviors.

In contrast, topics such as sun protection and skin cancer awareness demonstrated high baseline knowledge, likely reflecting prior public health exposure. However, improvements in attitudes toward sunscreen use despite this ceiling effect highlight an important distinction between knowledge and behavior. These findings suggest that even when adolescents are aware of preventive strategies, targeted education may still strengthen motivation and perceived value of these behaviors. This is consistent with prior evidence demonstrating that school-based interventions can effectively influence attitudes and behaviors regardless of baseline knowledge levels [7].

Attitudinal changes further support the effectiveness of the intervention. Increased agreement that sunscreen use is worthwhile and that acne is a normal part of development suggests greater alignment with preventive health behaviors and reduced stigma. The persistence of these changes at one-month follow-up indicates that these shifts were internalized rather than transient, reinforcing the potential for durable behavior change.

Improvements in self-efficacy and self-advocacy represent particularly meaningful outcomes, especially among a rural population facing barriers to dermatologic care. Participants demonstrated increased confidence in knowing where to seek help and greater comfort discussing concerns with healthcare providers. These improvements are particularly clinically relevant given the well-documented disparities in dermatology access, which contribute to delays in diagnosis and limited preventive education in underserved populations [3,4]. Similar patterns have been reported among rural high school students, where a school-based health literacy intervention improved both health knowledge and self-efficacy [13]. Our findings extend this evidence to dermatologic health education, demonstrating improvements in adolescents’ confidence in managing and seeking help for skin concerns.

The high rate of self-reported behavior change at one-month follow-up supports that improvements in knowledge, attitudes, and self-efficacy translated into substantive improvements in everyday health practices. Nearly nine out of 10 participants reported adopting at least one new skin care behavior, with sun-protective practices reported most frequently. Many responses described incorporating these behaviors into daily routines, highlighting the early development of sustainable preventive habits.

The observed progression from improved knowledge and attitudes to increased self-efficacy and behavior change is consistent with established health behavior frameworks, including the Health Belief Model. This framework suggests that individuals are more likely to adopt preventive behaviors when they recognize the benefits of those behaviors and feel confident in their ability to perform them [14]. Rather than reflecting knowledge acquisition alone, the findings of the present study suggest that addressing multiple behavioral determinants may be important for promoting sustained skin health practices among adolescents. Although the intervention was not explicitly designed around this framework, these findings provide additional support for educational approaches that address both knowledge and behavioral factors to encourage preventive skin health behaviors. 

Together, these findings suggest that school-based skin health interventions can improve not only dermatologic knowledge but also the attitudes, confidence, and health behaviors that support long-term preventive care. By addressing knowledge gaps, reinforcing positive health beliefs, and fostering self-efficacy, these interventions may empower adolescents to take a more active role in managing their skin health, particularly in rural and medically underserved communities.

Limitations

Several limitations should be considered when interpreting these findings. Outcomes were based on self-reported survey data, including open-ended reflections, which are subject to recall and social desirability bias, particularly for behavior change measures. The investigator-developed survey did not undergo formal validation or testing for reliability, which may limit the validity and generalizability of the measured outcomes. Participant attrition occurred across study time points, with fewer students completing the one-month follow-up survey than the baseline assessment. Although common in school-based intervention studies, this may have introduced selection bias if participants who completed follow-up differed systematically from those who did not. 

The study lacked a control or comparison group, so observed changes cannot be attributed exclusively to the educational intervention. Furthermore, the study was conducted at a single rural school, which may limit the generalizability of the findings to other populations or geographic regions. Several knowledge items demonstrated high baseline performance, resulting in ceiling effects that limited the ability to detect statistically significant improvements. This likely reflects the inclusion of students enrolled in the Career Pathway Academy of Health Sciences, who may have entered the study with greater baseline knowledge and interest in health-related topics. Finally, the follow-up period was limited to one month and therefore may not reflect the long-term sustainability of observed knowledge gains and behavior changes. 

Despite these limitations, the study provides evidence that a school-based skin health intervention can improve dermatologic knowledge, attitudes, self-efficacy, and self-reported behaviors among rural adolescents.

## Conclusions

This study demonstrates that a brief, school-based, medical student-led skin health education intervention can improve dermatologic knowledge, attitudes, self-efficacy, and preventive health behaviors among rural adolescents. By improving dermatologic knowledge while fostering positive attitudes and self-efficacy, this intervention may help translate learning into meaningful preventive health behaviors. These findings support the role of medical student-led skin health education programs in improving skin health literacy and empowering adolescents to take an active role in their health, particularly in rural and medically underserved communities.

## Appendices

Appendix A

Questions on All Three Survey Time Points

Demographics* *

Age

Gender

▢ Male ▢ Female ▢ Non-Binary ▢ Transgender ▢ Prefer not to say 

Grade
 

Prior exposure 

Have you learned about sun safety in school before?

▢ Yes ▢ No
 

Knowledge

What is the outermost layer of the skin called?

Epidermis 

Dermis 

Subcutaneous fat 

Muscle 

You should still wear sunscreen on cloudy days.

▢  True

▢  False 

Acne is caused only by poor hygiene.

▢  True

▢  False

What insects can spread diseases? Circle all that apply.

Mosquitoes 

Ticks 

Butterflies 

Ladybugs

Attitudes and beliefs* *

Taking care of my skin is important for my overall health

▢ Strongly Disagree ▢ Disagree ▢ Neutral ▢ Agree ▢ Strongly Agree

Wearing sunscreen is important to protect my skin

▢ Strongly Disagree ▢ Disagree ▢ Neutral ▢ Agree ▢ Strongly Agree

Acne is a normal part of growing up

▢ Strongly Disagree ▢ Disagree ▢ Neutral ▢ Agree ▢ Strongly Agree

I feel confident I can keep my skin safe when I am outdoors

▢ Strongly Disagree ▢ Disagree ▢ Neutral ▢ Agree ▢ Strongly Agree
 

Behaviors 

I wear sunscreen when I go outside 

▢ Never ▢ Rarely ▢ Sometimes ▢ Often ▢ Always

I wear protective clothing (hats, long sleeves, sunglasses) when I am outside 

▢ Never ▢ Rarely ▢ Sometimes ▢ Often ▢ Always

I avoid touching or picking at my skin 

▢ Never ▢ Rarely ▢ Sometimes ▢ Often ▢ Always

Self-Efficacy

I feel confident in how I take care of my skin

▢ Strongly Disagree ▢ Disagree ▢ Neutral ▢ Agree ▢ Strongly Agree

I know who to ask if I have questions about skin, hair, or nails 

▢ Strongly Disagree ▢ Disagree ▢ Neutral ▢ Agree ▢ Strongly Agree

I feel comfortable talking with a doctor or parent about skin concerns 

▢ Strongly Disagree ▢ Disagree ▢ Neutral ▢ Agree ▢ Strongly Agree

Question on the one-month follow-up survey

Open-Ended 

What is one thing you learned today about skin health? 
